# Supplementary material for: Hydroxychloroquine/chloroquine for the treatment of hospitalized patients with COVID-19: An individual participant data meta-analysis
Source: PLoS One. 2022 Sep 29;17(9):e0273526. doi: 10.1371/journal.pone.0273526 (PMC9521809; doi:10.1371/journal.pone.0273526)
Supplement: S3 Table — (PDF) [file pone.0273526.s006.pdf]

S3 Table. Changes to the Prespecified Statistical Analysis Plan

When the change was made	Change	Reason
After examining baseline covariates and outcome missingness, but not outcome data themselves	We decided to extend the outcome definition from day 28-30 post-enrollment to day 28-35 post-enrollment. A patient's outcome was taken to be their earliest recorded ordinal score between day 28 and day 35 post-enrollment (inclusive).	Missingness in the outcome measurements. The change increased the number of patients with valid outcome values from 90.3% to 95.3%.
	We decided to use a simple count of those comorbidities without significant missingness in place of a weighted Charlson (or Charlson-like) comorbidity score.	Missingness in baseline comorbidity indicators; not having requested standard Charlson indicators from each site.
	We simplified the safety variables under consideration.	Extensive missingness in QTc and elevated LFTs AE/SAE results.
	We modified the form of our prespecified regression model for the primary outcome.	Following establishing the total sample size and simulating outcome data from the empirical distribution of baseline patient characteristics.
After examining/analyzing the outcome data	We decided to use superpopulation rather than finite sample standardized estimators of treatment effect for our primary outcome analysis.	For three reasons: (1) the uncertainty in the superpopulation estimator is more directly comparable to that of the maximum likelihood estimator; (2) the finite-sample estimator requires assumptions about the dependence between individual-level potential outcomes; and (3) our choice of assumption—to treat the potential outcomes as independent—potentially made the associated uncertainty intervals misleadingly narrow.
	We set as missing BMIs less than 10 and greater than 70; for the outcome analysis, these were imputed in the same step as the other baseline covariates using multiple imputation.	Extremeness of these values.
	We decided not to fit a category-specific ordinal model as a sensitivity analysis.	Time and effort; the reasonable within-sample fit of the simpler models.

When the change was made	Change	Reason
	We decided to de-emphasize our pre-specified conditional effect measure (relative risk of mechanical ventilation/ECMO or death).	The associated uncertainty intervals were extremely wide, perhaps due to the flexibility of our prespecified model.
	We decided to include model-standardized estimates of the risk difference for mortality, both overall and by subgroup.	This was considered informative and straightforward, given the model. Additionally, risk differences are considered a more interpretable measure of subgroup effects than odds ratios because of their collapsibility [1,2].
	We decided to include a subgroup analysis based on quintiles of a baseline risk score.	Following recommendations of Kent et al [3].
	We decided not to examine whether site × treatment interactions are associated with site-level covariates or individual-level covariates averaged within sites.	There was very little variation in the estimated site × treatment interactions.
	We replaced mortality at day 28-30 as a safety outcome with mortality at day 28-35 as a secondary outcome, and conducted an analysis of this parallel to that of our primary outcome.	We had prespecified all-cause mortality at or before day 28/30 as a safety outcome. However, we judged that an analysis of mortality parallel to that of our primary outcome would be clinically relevant.
	In the primary outcome and mortality analyses, we treated 6 extreme BMI values (<10 or >70) as missing.	We suspected that these values were mistaken or could bias our results, and were unable to definitively establish their accuracy.
	We added an exploratory post-hoc subgroup analysis based on time between symptom onset and enrollment.	Suggested in review.

References

1. Didelez V, Stensrud MJ. On the logic of collapsibility for causal effect measures. Biom J. 2021;64: 235-242. doi:10.1002/bimj.202000305
2. Greenland S. Noncollapsibility, confounding, and sparse-data bias. Part 1: the oddities of odds. J Clin Epidemiol. 2021;138: 178-181. doi:10.1016/j.jclinepi.2021.06.007
3. Kent DM, Paulus JK, van Klaveren D, D'Agostino R, Goodman S, Hayward R, et al. The Predictive Approaches to Treatment effect Heterogeneity (PATH) statement. Ann Int Med. 2020;172: 35-45. doi:10.7326/M18-3667
