# Supplementary material for: Hydroxychloroquine/chloroquine for the treatment of hospitalized patients with COVID-19: An individual participant data meta-analysis
Source: PLoS One. 2022 Sep 29;17(9):e0273526. doi: 10.1371/journal.pone.0273526 (PMC9521809; doi:10.1371/journal.pone.0273526)
Supplement: S2 Fig — Conditional effects from the Bayesian proportional odds model. Shown are (1) the relative risk of mechanical ventilation or death at day 28–35; (2) the estimated probabilities of mechanical ventilation or death at day 28–35 under control and HCQ/CQ; and (3) the log proportional odds ratio comparing HCQ/CQ and control. Each of these effects are shown for reference individuals with the following covariate values: age 60, BMI 25, no baseline comorbidities, baseline ordinal score of 5, and sex coefficient set between male and female values. Curves for continuous covariates are accompanied by 50% and 95% credible bands; intervals for discrete covariates are accompanied by 66% and 95% credible intervals. BMI indicates body mass index; HCQ/CQ, hydroxychloroquine or chloroquine. (PDF) [file pone.0273526.s011.pdf]

# S2 Fig. Conditional Covariate Effects

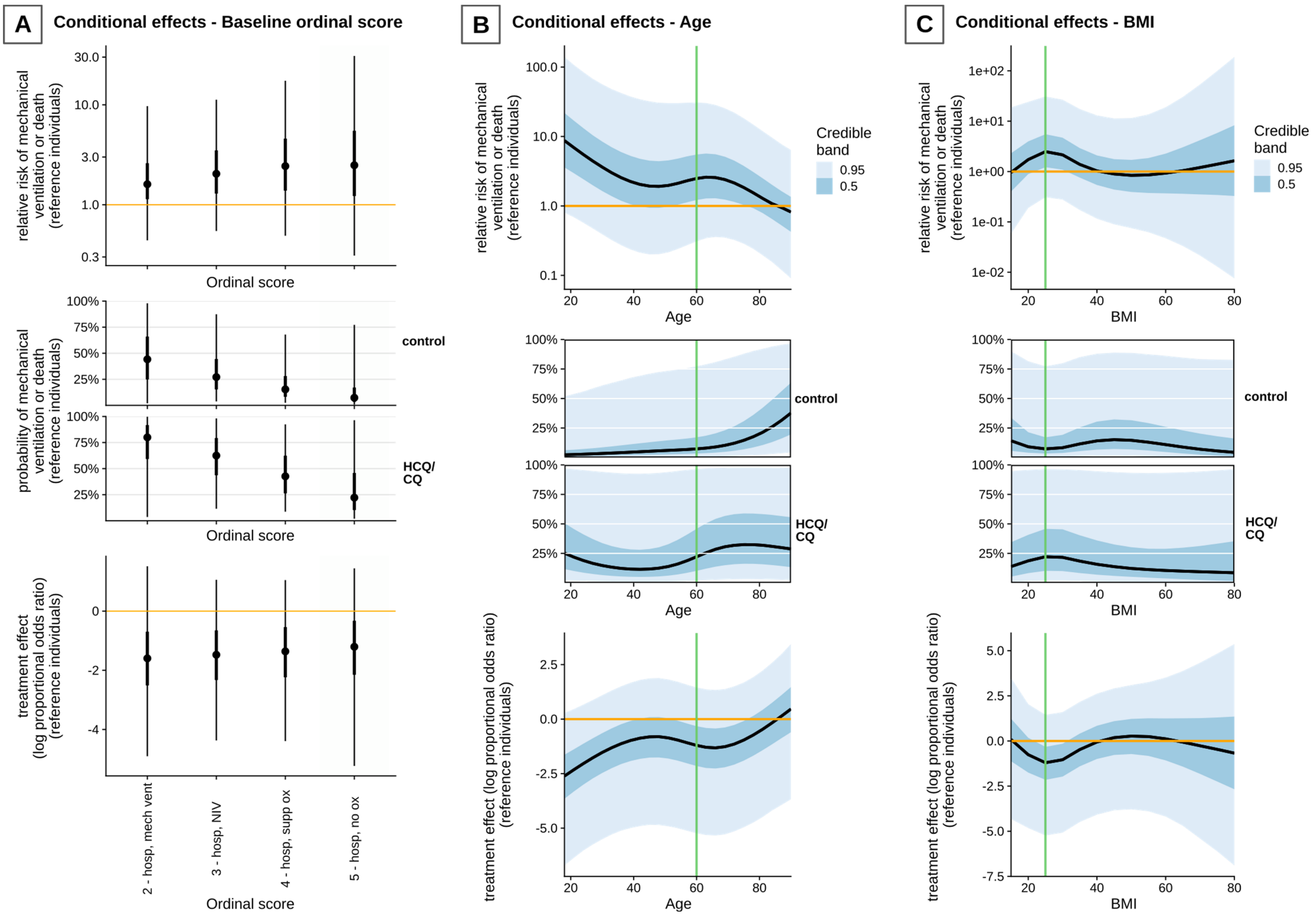

## *S2 Fig (continued)*

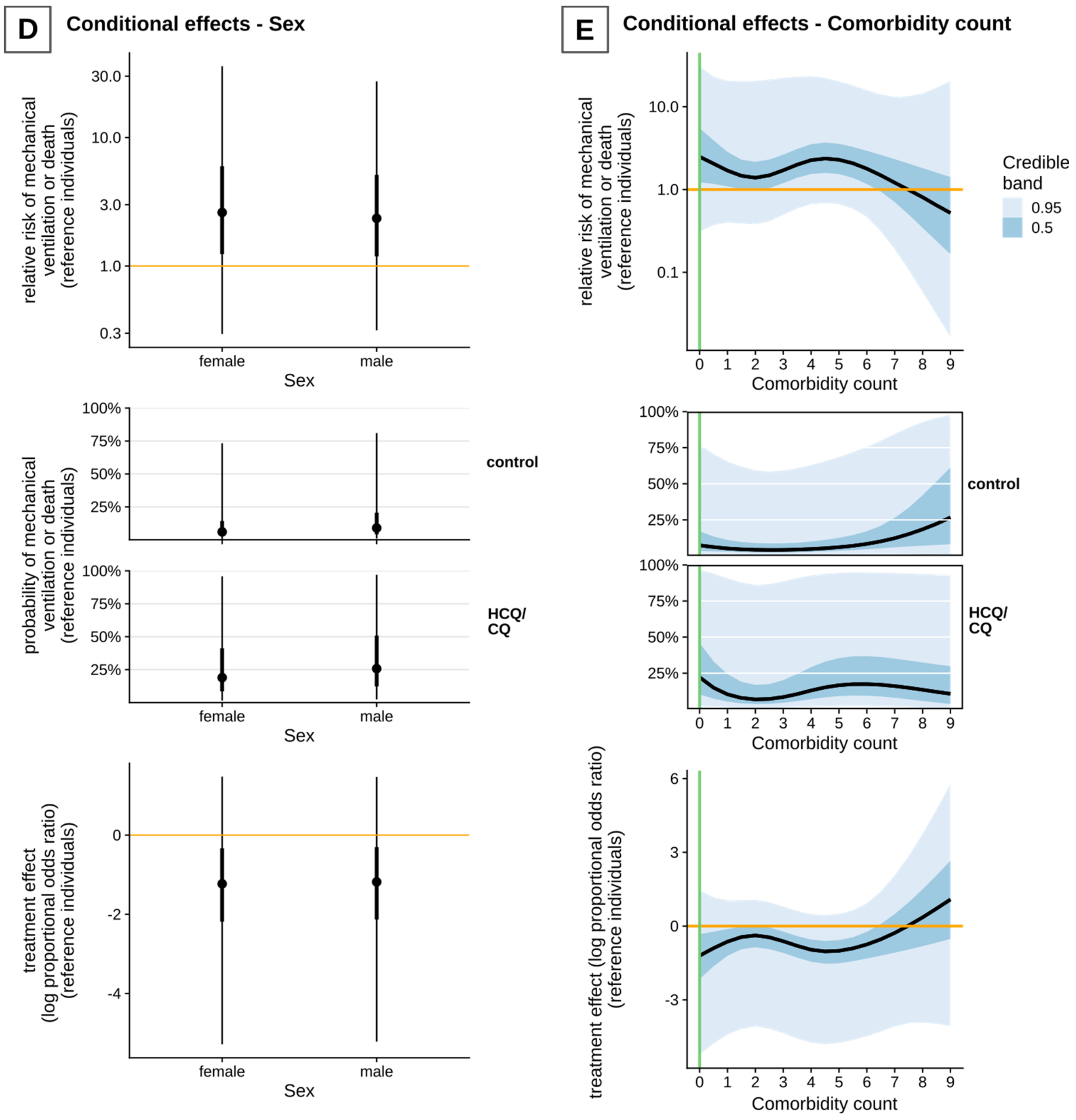


Conditional effects from the Bayesian proportional odds model. Shown are (1) the relative risk of mechanical ventilation or death at day 28-35; (2) the estimated probabilities of mechanical ventilation or death at day 28-35 under control and HCQ/CQ; and (3) the log proportional odds ratio comparing HCQ/CQ and control. Each of these effects are shown for reference individuals with the following covariate values: age 60, BMI 25, no baseline comorbidities, baseline ordinal score of 5, and sex coefficient set between male and female values. Curves for continuous covariates are accompanied by 50% and 95% credible bands; intervals for discrete covariates are accompanied by 66% and 95% credible intervals.

BMI indicates body mass index; HCQ/CQ, hydroxychloroquine or chloroquine.
