# Supplementary material for: Hydroxychloroquine/chloroquine for the treatment of hospitalized patients with COVID-19: An individual participant data meta-analysis
Source: PLoS One. 2022 Sep 29;17(9):e0273526. doi: 10.1371/journal.pone.0273526 (PMC9521809; doi:10.1371/journal.pone.0273526)
Supplement: S3 Fig — Shown are both plug-in estimates (based on the proportion of deaths in each subgroup) along with 95% CIs, and model-adjusted estimates with 95% credible intervals. The model used is the same as for the primary outcome analysis. HCQ/CQ indicates hydroxychloroquine or chloroquine. (PDF) [file pone.0273526.s012.pdf]

S3 Fig. Estimated Mortality Rate in Subgroups Under Both Control and HCQ/CQ

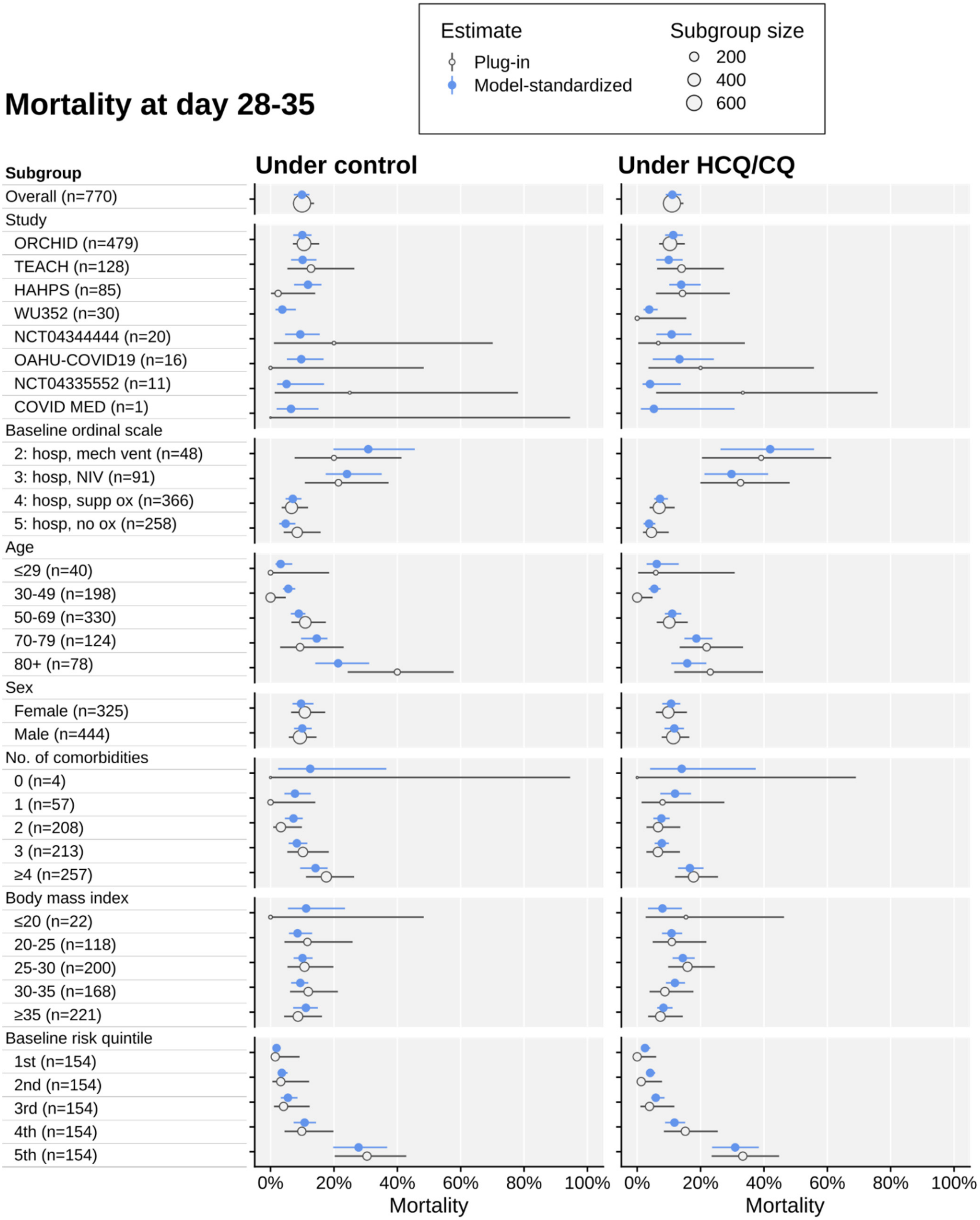

Shown are both plug-in estimates (based on the proportion of deaths in each subgroup) along with 95% CIs, and model-adjusted estimates with 95% credible intervals. The model used is the same as for the primary outcome analysis.

HCQ/CQ indicates hydroxychloroquine or chloroquine.
