# Supplementary material for: Hydroxychloroquine/chloroquine for the treatment of hospitalized patients with COVID-19: An individual participant data meta-analysis
Source: PLoS One. 2022 Sep 29;17(9):e0273526. doi: 10.1371/journal.pone.0273526 (PMC9521809; doi:10.1371/journal.pone.0273526)
Supplement: Supplementary file 11 [file pone.0273526.s004.pdf]

S1 Table. Data Dictionary from Data Harmonization Spreadsheet

In addition to the variables below, we requested inclusion and exclusion criteria from each trial.

Variable	Format	Description and comments
Patient ID	(Study-specific format)	
Other ID(s)	(Study-specific format)	
Treatment group	(Study-specific format)	
Enrollment date (year)	YYYY	Year of enrollment
Enrollment date (month)	Numeric (1-12)	Month of enrollment
Symptom onset date	Numeric (days)	Enrollment date - symptom onset date
Screening date	Numeric (days)	Enrollment date - screening date
Admission date	Numeric (days)	Enrollment date - admission date
Randomization date	Numeric (days)	Randomization date - enrollment date
Date of first dose	Numeric (days)	Date of first dose - date of enrollment (numeric; days)
Date of last dose	Numeric (days)	Date of last dose - date of enrollment
Ordinal outcome scale	Ordinal scale<br>1 = Death<br>2 = Hospitalized, on invasive mechanical ventilation or extracorporeal membrane oxygenation (ECMO)<br>3 = Hospitalized, on non-invasive ventilation or high-flow oxygen devices<br>4 = Hospitalized, requiring supplemental oxygen<br>5 = Hospitalized, not requiring supplemental oxygen<br>6 = Not hospitalized, limitation on activities<br>7 = Not hospitalized, no limitations on activities	Baseline ordinal scales as well as all available through day 30 post enrollment (Day 0, Day 7, Day 14, Day 28/30 are most important)
Date at which ordinal scale is measured	Numeric (days)	Date of ordinal outcome status – date of enrollment
Hospitalization length of stay	Numeric (days)	Duration in days between enrollment and day 28/30 post enrollment
Duration of mechanical ventilation	Numeric (days)	Duration in days between enrollment and day 28/30 post enrollment
All-cause mortality	1 = Yes, 0 = No	Between enrollment and day 28/30 post enrollment
All-cause mortality – date of death	Numeric (days)	Between enrollment and day 28/30 post enrollment
Number of AEs	Numeric (count)	Between enrollment and day 28/30 post enrollment
Number of SAEs	Numeric (count)	Between enrollment and day 28/30 post enrollment
Number of AEs for QTC Prolongation	Numeric (count)	Between enrollment and day 28/30 post enrollment

Variable	Format	Description and comments
Number of SAEs for QTC Prolongation	Numeric (count)	Between enrollment and day 28/30 post enrollment
Number of AEs for elevated liver function test	Numeric (count)	Between enrollment and day 28/30 post enrollment
Number of SAEs for elevated liver function test	Numeric (count)	Between enrollment and day 28/30 post enrollment
Number of AEs for arrhythmia or cardiac arrest	Numeric (count)	Between enrollment and day 28/30 post enrollment
Number of SAEs for arrhythmia or cardiac arrest	Numeric (count)	Between enrollment and day 28/30 post enrollment
Age	Numeric (years)	Age rounded *down* to beginning of 5-year bracket – e.g., 43 → 40; 39 → 35
Sex	1 = Male, 2 = Female	
Race	1 = American Indian/Alaska Native;<br>2 = Asian;<br>3 = Black/African American;<br>4 = Native Hawaiian/Pacific Islander;<br>5 = White;<br>6 = Multiple;<br>7 = Other/declined;<br>8 = Unknown/unavailable	
Ethnicity	0 = Not of Hispanic, Latinx, or Spanish origin<br>1 = Hispanic, Latinx, or Spanish origin<br>2 = Unknown	
BMI	numeric	
On mechanical ventilation at enrollment	1 = Yes, 0 = No	
AIDS (do not include HIV-positive without AIDS criteria)	1 = Yes, 0 = No	
Cerebrovascular disease	1 = Yes, 0 = No	
A prior myocardial infarction	1 = Yes, 0 = No	
Congestive heart failure	1 = Yes, 0 = No	
Dementia	1 = Yes, 0 = No	
COPD	1 = Yes, 0 = No	
Asthma	1 = Yes, 0 = No	
History of hypertension	1 = Yes, 0 = No	
HIV positive (without AIDS)	1 = Yes, 0 = No	
Solid tumor	1 = Yes, 0 = No	
Liver disease	1 = Yes, 0 = No	
Diabetes mellitus	1 = Yes, 0 = No	
Cigarette or tobacco smoking	1 = Yes, 0 = No	
Vaping	1 = Yes, 0 = No	
Concurrent corticosteroid use	1 = Yes, 0 = No	
Concurrent azithromycin use	1 = Yes, 0 = No	
