# Supplementary material for: Hydroxychloroquine/chloroquine for the treatment of hospitalized patients with COVID-19: An individual participant data meta-analysis
Source: PLoS One. 2022 Sep 29;17(9):e0273526. doi: 10.1371/journal.pone.0273526 (PMC9521809; doi:10.1371/journal.pone.0273526)
Supplement: Supplementary file 14 [file pone.0273526.s009.pdf]

S6 Table. Merging Trial Arms

Trial	Treatment groups	Treatment days	Treatment or control?	Dose by treatment group
ORCHID	2	5 days	T	HCQ: Day 1 400 mg twice daily; Days 2-5 200 mg twice daily
			C	Placebo
WU352	4	5 days	T	HCQ: Day 1 400 mg twice daily; Days 2-5 200 mg twice daily
			T	HCQ + AZM: HCQ: Day 1 400 mg twice daily; Days 2-5 200 mg twice daily; AZM: Day 1 500 mg once; Days 2-5 250 mg once daily
			T	CQ: Day 1 1000 mg once followed by 500 mg in 12 hours; Days 2-5 500 mg twice daily
			T	CQ + AZM: CQ: Day 1 1000 mg once followed by 500 mg in 12 hours; Days 2-5 500 mg twice daily; AZM: Day 1 500 mg once; Days 2-5 250 mg once daily
NCT04335552 (Duke University)	2	5 days	C	Arm 1: Supportive care alone
			T	Arm 2: Supportive care plus HCQ (Day 1 800 mg once; Days 2-5 600 mg once daily)
			C	Arm 3: Supportive care plus azithromycin (Day 1 500 mg once; Days 2-5 250 mg once daily)
			T	Arm 4: Supportive care plus HCQ (Day 1 800 mg once; Days 2-5 600 mg once daily) plus azithromycin (Day 1 500 mg once; Days 2-5 250 mg once daily)
TEACH	2	5 days	T	HCQ: Day 1 400 mg twice daily; Days 2-5 200 mg twice daily
			C	Placebo
COVID MED	4 (only Arms 2 and 4 included herein)	14 days	C	Arm 1: Standard care and lopinavir/ritonavir: Dosing: 400 mg/100 mg twice daily for 5-14 days
			T	Arm 2: Standard care and HCQ: Day 1 400 mg twice daily; Days 2-5 200 mg twice daily
			C	Arm 3: Standard care and losartan: Losartan 25 mg daily for 5-14 days; placebo daily for 5-14 days to replicate/control for twice-daily dosing
			C	Arm 4: Standard care and placebo: Placebo twice daily for 5-14 days
HAHPS	2	5 days	T	HCQ: Day 1 400 mg twice daily; Days 2-5 200 mg twice daily
			C	AZM: Day 1 500 mg once; Days 2-5 250 mg once daily
NCT04344444 (University Medical Center New Orleans)	3	5 days	C	Control: Supportive Care Only
			T	HCQ: Day 1 400 mg twice daily; Days 2-5 200 mg twice daily
			T	HCQ + AZM: HCQ: Day 1 400 mg twice daily; Days 2-5 200 mg twice daily; AZM: Day 1 500 mg once; Days 2-5 250 mg once daily
OAHU-COVID19	2	5 days	T	HCQ: Day 1 400 mg twice daily; Days 2-5 200 mg twice daily
			C	Usual care
