# Supplementary material for: Hydroxychloroquine/chloroquine for the treatment of hospitalized patients with COVID-19: An individual participant data meta-analysis
Source: PLoS One. 2022 Sep 29;17(9):e0273526. doi: 10.1371/journal.pone.0273526 (PMC9521809; doi:10.1371/journal.pone.0273526)
Supplement: Attachment — Submitted filename: Decision letterr.docx [file pone.0273526.s015.docx]

Dear Editor,

Thank you for choosing me as reviewer of your prestigious journal.

I read the manuscript carefully. It’s a well-written and robust manuscript, there are only some minor comments.

Minor comments:

1-Please add reference for PRISMA guide line.

2- the authors claimed that their study stick to PRISMA guide line. However, based on this protocol, search strategy should be presented in text or supplementary file. Also, PRISMA check list is necessary.

I read this manuscript several time. Its comprehensive and well-written, I have no major comment about methodology, data reporting and analysis. My suggestion is acceptance or minor revision.
